# Cardioprotective Action of *Ginkgo biloba* Extract against Sustained β-Adrenergic Stimulation Occurs via Activation of M_2_/NO Pathway

**DOI:** 10.3389/fphar.2017.00220

**Published:** 2017-05-11

**Authors:** Thássio R. R. Mesquita, Itamar C. G. de Jesus, Jucilene F. dos Santos, Grace K. M. de Almeida, Carla M. L. de Vasconcelos, Silvia Guatimosim, Fabrício N. Macedo, Robervan V. dos Santos, José E. R. de Menezes-Filho, Rodrigo Miguel-dos-Santos, Paulo T. D. Matos, Sérgio Scalzo, Valter J. Santana-Filho, Ricardo L. C. Albuquerque-Júnior, Rose N. Pereira-Filho, Sandra Lauton-Santos

**Affiliations:** ^1^Department of Physiology, Federal University of SergipeSão Cristóvão, Brazil; ^2^Departments of Physiology and Biophysics, Federal University of Minas GeraisBelo Horizonte, Brazil; ^3^Technology and Research Institute, Tiradentes UniversityAracaju, Brazil

**Keywords:** *Ginkgo biloba*, cardiac hypertrophy, chronic β-adrenergic stimulation, cholinergic signaling, endothelial nitric oxide synthase

## Abstract

*Ginkgo biloba* is the most popular phytotherapic agent used worldwide for treatment of several human disorders. However, the mechanisms involved in the protective actions of *Ginkgo biloba* on cardiovascular diseases remain poorly elucidated. Taking into account recent studies showing beneficial actions of cholinergic signaling in the heart and the cholinergic hypothesis of *Ginkgo biloba*-mediated neuroprotection, we aimed to investigate whether *Ginkgo biloba* extract (GBE) promotes cardioprotection *via* activation of cholinergic signaling in a model of isoproterenol-induced cardiac hypertrophy. Here, we show that GBE treatment (100 mg/kg/day for 8 days, v.o.) reestablished the autonomic imbalance and baroreflex dysfunction caused by chronic β-adrenergic receptor stimulation (β-AR, 4.5 mg/kg/day for 8 days, i.p.). Moreover, GBE prevented the upregulation of muscarinic receptors (M_2_) and downregulation of β_1_-AR in isoproterenol treated-hearts. Additionally, we demonstrated that GBE prevents the impaired endothelial nitric oxide synthase activity in the heart. GBE also prevented the pathological cardiac remodeling, electrocardiographic changes and impaired left ventricular contractility that are typical of cardiac hypertrophy. To further investigate the mechanisms involved in GBE cardioprotection *in vivo*, we performed *in vitro* studies. By using neonatal cardiomyocyte culture we demonstrated that the antihypertrophic action of GBE was fully abolished by muscarinic receptor antagonist or NOS inhibition. Altogether, our data support the notion that antihypertrophic effect of GBE occurs *via* activation of M_2_/NO pathway uncovering a new mechanism involved in the cardioprotective action of *Ginkgo biloba*.

## Introduction

*Ginkgo biloba* extract (GBE) has been recognized in the traditional Chinese medicine for its various therapeutic actions. GBE is composed of several components such as bilobalide, ginkgolide A, ginkgolide B, and ginkgolide C, which are responsible for its multiple pharmacological effects (Yoshikawa et al., 1999). However, although *G. biloba* leaves are widely prescribed as alternative herbal medicine for memory improvement, as well as for dementia (Tan et al., 2015), the precise mechanism by which *G. biloba* elicits neuroprotective and cognitive-enhancing effects remains to be determined. Notably, the cholinergic hypothesis, associated with augmented yield of the neurotransmitter acetylcholine (ACh), has been consistently reported as the primary mechanism of the beneficial pharmacological properties of *G. biloba* (Nathan, 2000; Kehr et al., 2012).

Although the benefits of GBE on neurological disorders are well known (Nathan, 2000; Tan et al., 2015), GBE has also been used for treatment of several cardiovascular diseases (Zhou et al., 2004; Kuller et al., 2010). However, most of the studies have attributed the cardioprotection of GBE to enhanced antioxidant activity (Pietri et al., 1997; Liebgott et al., 2000; Wang et al., 2016). Accordingly, augmented endogenous antioxidant enzymes mediated by GBE treatment led to a protective effect against acute myocardial infarction and injury caused by ischemia-reperfusion (Trumbeckaite et al., 2007; Panda and Naik, 2008). Moreover, the anti-inflammatory and antiapoptotic actions of GBE have been also involved in the attenuation of doxorubicin-induced cardiac injury (Liu et al., 2008). Altogether, these studies support the notion that GBE might be a promising cardiac agent against various pathological stimuli.

Despite the extensive body of evidence supporting the beneficial cardiac actions of GBE, the mechanisms involved are still unclear. Nitric oxide (NO) signaling has been consistently reported as a unique modulator of cardiovascular system under physiological and pathophysiological conditions (Barouch et al., 2002; Umar and van der Laarse, 2009). Accordingly, endothelial nitric oxide synthase (eNOS)-overexpressing mice display attenuated isoproterenol-induced cardiac hypertrophy (Ozaki et al., 2002), while eNOS knockout mice show a higher incidence of early afterdepolarization events associated with contractile dysfunction (Barouch et al., 2002; Wang et al., 2008). Moreover, impaired eNOS activity has been found in many forms of diseases such as hypertension (Chou et al., 1998), cardiac hypertrophy (Ozaki et al., 2002; Champion et al., 2004), myocardial infarction and ischemia-reperfusion injury (du Toit et al., 2007; Nakata et al., 2008), and heart failure (Couto et al., 2015). Notably, GBE has been also shown to enhance eNOS activity and, consequently, NO bioavailability in human endothelial cells (Koltermann et al., 2007). In line with these findings, GBE causes vasodilation on different vascular beds (Satoh and Nishida, 2004), being also involved in the restoration of impaired endothelial-dependent vasodilation in hypertensive rats (Kubota et al., 2006; Koltermann et al., 2007). Recently, the cardioprotective actions of GBE against adriamycin-induced acute cardiotoxicity have been reported by regulation of inflammatory and NO signaling pathways (El-Boghdady, 2013).

Thus, in spite of many clues indicating that GBE may be therapeutically relevant by balancing NO production, its involvement on the antihypertrophic effect of GBE is not yet reported. Therefore, based on the above considerations, the present study aimed to evaluate whether *G. biloba* promotes cardioprotection in a model of isoproterenol-induced cardiac hypertrophy and the mechanisms involved in these effects.

## Materials and Methods

### Animals

Male Wistar rats (250–270 g) were obtained from the Animal Care Facility of Federal University of Sergipe and maintained under a controlled 12-h light/12-h dark cycle at room temperature (23 ± 2°C). All experimental procedures were previously approved by the Ethics Committee for Animal Research of the Federal University of Sergipe, Brazil (Protocol #36/10).

### Experimental Groups

Animals were randomly divided into four experimental groups and the treatments were performed for 8 days: CTR, received saline (i.p. daily); ISO, received isoproterenol (4.5 mg/kg/day, i.p.); GBE, received *G. biloba* extract (100 mg/kg/day, v.o.) plus saline (i.p.); ISO + GBE, received isoproterenol (4.5 mg/kg/day, i.p.) plus *G. biloba* extract (100 mg/kg/day, v.o.). Isoproterenol was used as a non-specific β-adrenergic receptor (β-AR) agonist to induce cardiac hypertrophy, as previously described (Gavioli et al., 2014). The GBE used in this study was a standardized extract obtained from leaves, in which containing 25.5% Ginkgo flavonoids, 24.14% quercetin, 0.86% kaempferol, 0.55% isorhamnetin, and 6% terpenoids (ginkgolides and bilobalide). The GBE was provided by Fármacos (Sergipe) from Ningbo Traditional Chinese Pharmaceutical (China).

### Hemodynamic Measurements

The animals were anesthetized with thiopental sodium (50 mg/kg, i.p.) and a polyethylene catheter was implanted into the femoral artery. The catheter was tunneled into the back of the rats and exteriorized in the nape. After 24 h, the catheter was connected to a pressure transducer (FE221, Bridge Amp, ADInstruments, Bella Vista, NSW, Australia) coupled to a pre-amplifier (Powerlab 8/35, ADInstruments). Blood pressure and heart rate (HR) were recorded for 30 min and processed using a dedicated software (LabChart 7 Pro, ADInstruments).

### Autonomic Evaluation

Cardiac autonomic balance was evaluated by frequency domain (Macedo et al., 2016). To perform this analysis, the CardioSeries v2.4 software was used^1^. First, the beat-by-beat series obtained from pulsate arterial pressure recordings and HR were converted to data points every 100 ms using cubic spline interpolation (10 Hz). The interpolated series were divided into half-overlapping sequential sets of 512 data points (51.2 s). Before calculation of the spectral power density, the segments were visually inspected and the non-stationary data were not taken into consideration. The spectrum was calculated using the Fast Fourier Transformation algorithm and Hanning window was used to attenuate side effects. The spectrum is composed by bands of low frequency (LF; 0.2–0.75 Hz) and high frequency (HF; 0.75–3 Hz), being the results expressed as normalized units (nu), by calculating the percentage of the LF and HF variability with respect to the total power after subtracting the power of the very LF component (VLF frequencies < 0.20 Hz), namely LF/HF ratio. HF indicates the cardiac parasympathetic activity, while LF is an index of cardiac sympathetic activity and LF/HF ratio represents the sympatho-vagal balance to the heart.

The baroreflex sensitivity (BRS) was measured in the time domain by the sequence method (Bertinieri et al., 1985). Sequences of at least four heart beats with increased SAP followed by pulse interval lengthening or subsequent decrease of SAP with pulse interval shortening with correlation greater than 0.85 were identified as baroreflex sequence. The slope of the linear regression between SAP and pulse interval was considered as a measure of BRS (mmHg/s).

### Electrocardiography (ECG) Records

The rats were anesthetized and kept in the supine position with spontaneous breathing. For surface ECGs recording, three stainless steel electrodes were subcutaneously implanted and ECG signals were amplified (HP7754A, HP7754B, Hewlett-Packard, Chicago, IL, USA), digitalized (DI-710, Windaq Pro, Dataq, Akron, OH, USA) and stored in a computer for off-line processing. In all of the experimental groups, HR, corrected QT interval (QTc), QRS complex duration, and intrinsicoid deflection (ID) were measured in 10 consecutive beats. QT interval was corrected by HR using Bazett’s equation.

### Measurements of Left Ventricular Developed Pressure (LVDP) and Coronary Pressure

After 15 min of heparin administration (1,000 I.U., i.p.), the heart was quickly removed and carefully mounted in an aortic perfusion system (Langendorff technique) on a constant flow (8 mL/min) (Milan Peristaltic Pump, Paraná, Brazil). Then, the heart was perfused with Krebs solution (in mM: 118.0 NaCl, 4.7 KCl, 1.2 MgSO_4_, 25.0 NaHCO_3_, 1.8 CaCl_2_, 11.1 glucose, 1.2 KH_2_PO_4_; pH was adjusted to 7.4) that had been previously filtered through a cellulose acetate membrane (0.45 μm), oxygenated (95% O_2_ + 5% CO_2_) and kept at 37 ± 0.1°C (Haake F3, Berlin, Germany). The left intraventricular pressure was measured using a water-filled balloon introduced into the cavity of the left ventricle. This device was coupled to a pressure transducer (HP 1290A, Hewlett-Packard, Chicago, IL, USA). Signals were amplified (HP7754A, HP7754B), digitized (DI-710, Windaq Pro, Dataq, Akron, OH, USA) and stored in a computer. The system was calibrated using a mercury column. Coronary pressure was measured at the tip of the aortic cannula and monitored in a water column.

### Western Blot Analyses

Western blots were performed as previously described (Mota et al., 2015), with some modifications. Thirty to fifty microgram of protein were resolved on SDS–PAGE, transferred to a PVDF membrane, and incubated with the following primary antibodies: anti-eNOS (1:1000, sc-654), anti-nNOS (1:1000, sc-8309), M_2_ (1:1000, sc-9107) anti-peNOS^ser1177^ (1:1000, sc-12972), anti-pnNOS^ser852^ (1:1000, sc-19826), anti-SERCA2 (1:2500, sc-376235), and anti-GAPDH (1:3000, sc-32233) from Santa Cruz Biotechnology or β_1_-AR (1:1000, ab3442, Abcam). All of them incubated at 4°C overnight. After incubation with appropriate secondary peroxidase-coupled antibodies for 1 h, immunodetection was carried out using enhanced chemiluminescence (Amersham Biosciences) followed by densitometric analysis with software ImageJ. Protein levels were expressed as a ratio of optical densities. GAPDH was used as a control for any variations in protein loading.

### Histopathology Analyses

After experimental procedures, rats were anesthetized and euthanized by applying potassium chloride solution (KCl – 10%) into the jugular vein. The hearts were fixed in formalin (10%), embedded in paraffin and cut at 5 μm thickness followed by staining with hematoxylin-eosin. Morphometric analysis was performed using the software ImageJ. The mean nuclear area was extracted from each histological slide.

### Neonatal Cardiomyocytes Culture

Rat neonatal cardiomyocytes (3-days old) were cultured as previously described (Rocha-Resende et al., 2012). Briefly, cardiac cells were plated in dishes containing M199 medium supplemented with 100 units/mL penicillin, 100 μg/mL streptomycin, 10% fetal bovine serum, and 2 mM/L L-glutamine. To prevent growth of fibroblasts, medium was supplemented with 20 μg/mL cytosine-D-arabinofuranoside (ARA-c). After 48 h, neonatal cardiomyocytes were exposed to isoproterenol (10 μM) and/or GBE (100 μg/mL). When appropriated, cells were incubated with atropine (AT, 10 μM) or Nω-nitro-L-arginine methyl ester hydrochloride (L-NAME, 10 μM) for 48 h. Afterward, the cells were then used for immunofluorescence.

### Immunofluorescence

Neonatal cardiomyocytes were fixed in a 4% paraformaldehyde solution and permeabilized with 0.5% Triton-X100. After blocking, cardiomyocytes plated onto glass coverslips were incubated for 1 h at room temperature with Alexa Fluor 488-conjugated anti-phalloidin (1:100, A12379, Invitrogen). Nuclear staining was obtained by incubating with 4,6-diamidino-2-phenylindole (DAPI, 1:50). Surface area of cardiomyocytes was measured in phalloidin stained cells. Images were acquired with a Zeiss LSM 510 confocal system located at Center of Acquisition and Processing of Images (CAPI–ICB, UFMG). All images were representative of two independent experiments in which multiple cells were evaluated.

### Statistical Analyses

All data are expressed as mean ± SEM. Statistical comparisons were performed using GraphPad Prism 5.1 (San Diego, CA, USA). Normality and equality of variance were tested by Shapiro–Wilk and Levene test, respectively. Significant differences between groups were determined with one-way ANOVA followed by the Bonferroni *post hoc* test. Differences were considered to be statistically significant when *p* < 0.05.

## Results

### *Ginkgo biloba* Extract Restores the Autonomic Imbalance of Isoproterenol-Treated Rats

First, we show that ISO-treated rats (4.5 mg/kg/day for 8 days) developed prominent cardiac hypertrophy, indicated by an increase in heart weight to body weight or tibia length ratios compared with untreated rats. Remarkably, concomitant GBE treatment (100 mg/kg) prevented the cardiac hypertrophy induced by chronic β-AR stimulation (**Table 1**). *In vivo* evaluation of cardiovascular function demonstrated a decreased HR in ISO-treated rats, which was not prevented after GBE treatment. Despite a tendency toward lower mean arterial pressure in ISO group, there was no significant change when compared to untreated group (**Table 2**).

**Table 1:** Biometric parameters in all experimental groups.

	CTR	ISO	GBE	ISO + GBE
HW/BW (mg/g)	3.9 ± 0.01	5.3 ± 0.01^∗∗∗^	3.9 ± 0.02	4.4 ± 0.01^##^
HW/TL (mg/cm)	273 ± 0.01	327 ± 0.01^∗∗^	248 ± 0.01	276 ± 0.01^#^

*Data are represented as means ± SEM, (*n* = 14). ^∗∗^*p* < 0.01 and ^∗∗∗^*p* < 0.001 vs. CTR; ^*#*^*p* < 0.05 and ^*##*^*p* < 0.01 vs. ISO, one-way ANOVA followed by Bonferroni’s Test. Heart weight/body weight (HW/BW); Heart weight/tibia length (HW/TL).*

**Table 2:** *In vivo* hemodynamic measurements in all experimental groups.

	CTR	ISO	GBE	ISO + GBE
HR (BPM)	386 ± 16	325 ± 6.0^∗^	387 ± 12	332 ± 14
MAP (mmHg)	109 ± 1.5	100 ± 0.4	108 ± 3.2	111 ± 1.9

*Heart rate (HR); mean arterial pressure (MAP). Data are represented as means ± SEM, (*n* = 5). ^∗^*p* < 0.05 vs. ISO, one-way ANOVA followed by Bonferroni’s post-test.*

**FIGURE 1:**
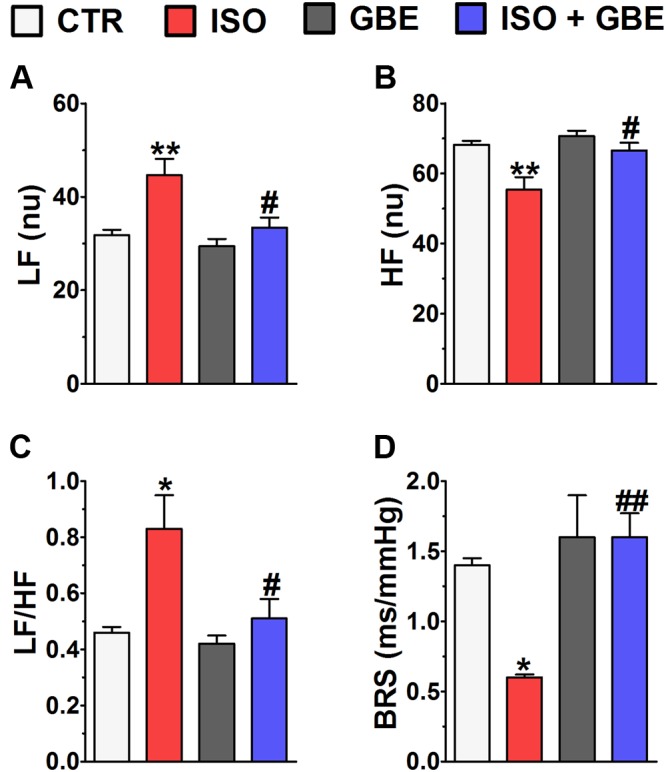
***Ginkgo biloba* extract restores the impaired cardiovascular autonomic modulation and baroreflex sensitivity of hypertrophic hearts. (A)** low frequency band (LF), **(B)** high frequency band (HF), **(C)** LF/HF ratio, and **(D)** baroreceptor reflex sensitivity (BRS). Data are represented as means ± SEM, (*n* = 5). ^∗^*p* < 0.05 and ^∗∗^*p* < 0.01 vs. CTR, ^#^*p* < 0.05 and ^##^p < 0.01 vs. ISO, one-way ANOVA followed by Bonferroni’s post-test.

It is well known that chronic β-AR stimulation induces profound alterations in autonomic nervous system (Shivkumar and Ardell, 2016), therefore we next evaluated whether GBE modulates the autonomic balance and BRS of hypertrophic hearts. As expected, chronic β-AR treatment led to a greater power in the LF band (**Figure 1A**) and smaller power in the HF band (**Figure 1B**) when compared to control, an indication of sympathovagal imbalance. Notably, GBE treatment fully restored sympathovagal balance in ISO-treated rats (LF/HF ratio, **Figure 1C**). Moreover, as shown in **Figure 1D** ISO-treated rats displayed decreased spontaneous BRS, which was rescued by GBE treatment. Altogether, our data indicate that GBE treatment modulates the sympathovagal balance of ISO-treated rats, suggesting the involvement of the cholinergic parasympathetic drive in the restoration of autonomic balance mediated by GBE.

### *Ginkgo biloba* Extract Prevents the Upregulation of Cardiac Muscarinic Receptor and Downregulation of β_1_-AR Induced by Chronic β-AR Stimulation

**FIGURE 2:**
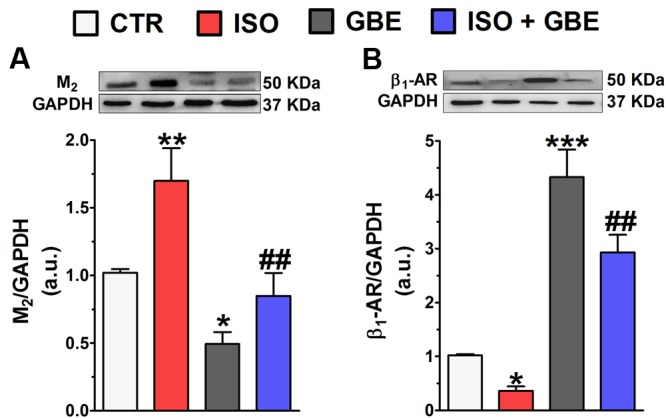
***Ginkgo biloba* extract modulates cardiac muscarinic receptor (M_2_) and β-adrenergic receptors (β_1_-AR) in hypertrophied hearts.** (**A,B**, top) Representative western blot and quantitative analysis of M_2_ **(A)** and β_1_-AR **(B)** protein levels. Data are represented as means ± SEM, (*n* = 3–6). ^∗^*p* < 0.05, ^∗∗^*p* < 0.01, and ^∗∗∗^*p* < 0.001 vs. CTR, ^##^*p* < 0.01 vs. ISO, one-way ANOVA followed by Bonferroni’s post-test.

To investigate whether GBE modulates expression levels of muscarinic receptor (M_2_) and β_1_-AR in the heart, we next performed the western-blot technique. As shown in the **Figure 2**, protein levels of M_2_ were upregulated (**Figure 2A**), while the expression of β_1_-AR was downregulated (**Figure 2B**) in left ventricles from ISO-treated rats when compared to control. On the other hand, GBE treatment alone led to a decreased expression of M_2_ (**Figure 2A**), while the protein levels of β_1_-AR was upregulated (**Figure 2B**). Importantly, GBE treatment was able to prevent alterations of M_2_ and β_1_-AR induced by ISO suggesting that under this condition appropriated autonomic balance was restored. Once again, this finding reinforces the idea that *G. biloba*-mediated cardioprotective actions involve the activation of cholinergic activity.

### *Ginkgo biloba* Extract Restores eNOS Protein Expression and Activity of Hypertrophic Hearts

**FIGURE 3:**
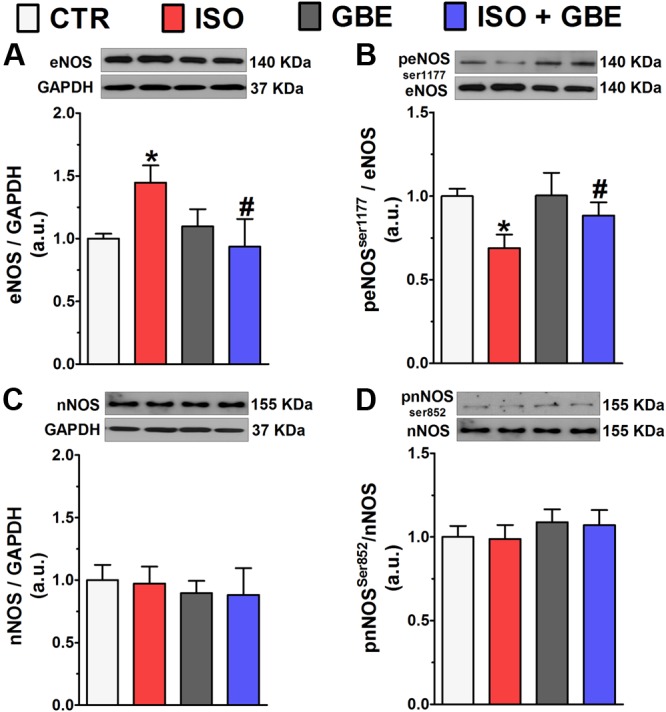
***Ginkgo biloba* extract restores endothelial nitric oxide synthase (eNOS) protein expression and activity of hypertrophic hearts.** (**A–D**, top) Representative western blot and quantitative analysis of eNOS **(A)**, peNOS^ser1177^**(B)**, nNOS **(C)**, and pnNOS^ser852^ **(D)** protein levels. Data are represented as means ± SEM, (*n* = 4–6). ^∗^*p* < 0.05 vs. CTR, ^#^*p* < 0.05 vs. ISO, one-way ANOVA followed by Bonferroni’s post-test.

It is well known that augmented local cholinergic activity leads to an increase in NO levels (Rocha-Resende et al., 2012). Therefore, in order to better understand whether the synthesis of NO is involved in cardioprotective actions of GBE, we next evaluated protein expression and activity of the constitutive NO synthesis (eNOS and nNOS) in the heart. As shown in the **Figure 3A**, ISO-treated rats presented an upregulation of eNOS, while its activity was markedly reduced (**Figure 3B**). In contrast, GBE treatment of ISO-treated rats fully restored eNOS levels and activity when compared to control. Importantly, neither nNOS expression nor activity was changed by ISO or GBE treatment (**Figures 3C,D**). Altogether, our data show that GBE treatment restores impaired eNOS.

### *Ginkgo biloba* Extract Prevents ISO Induced Hypertrophic Remodeling

**FIGURE 4:**
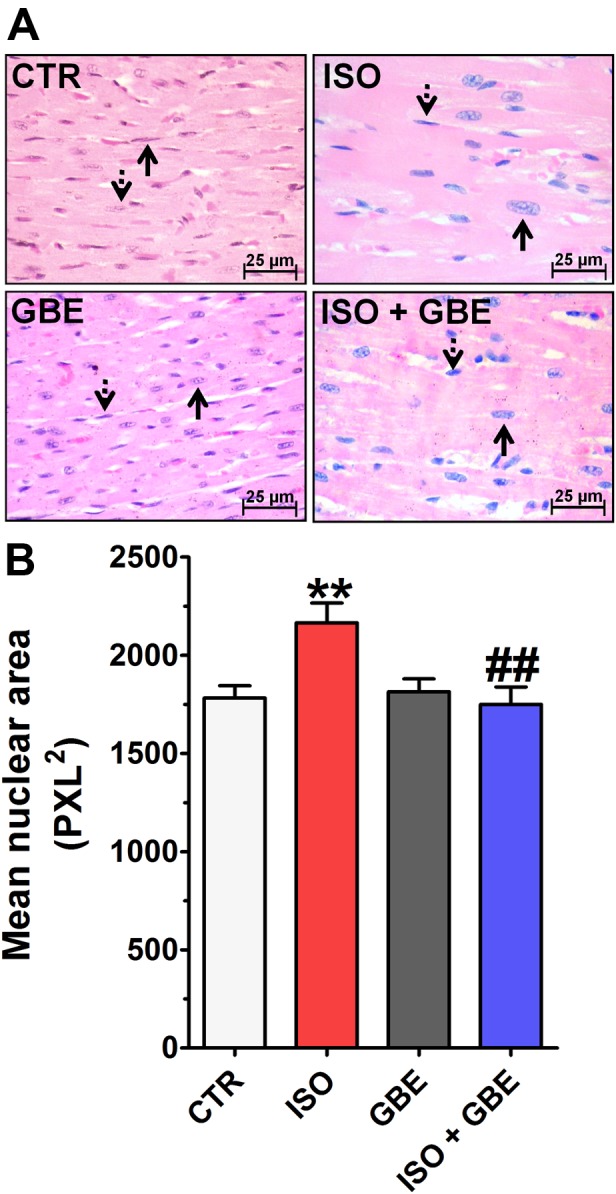
***Ginkgo biloba* extract prevents the development of cardiac hypertrophy induced by chronic β-adrenergic stimulation. (A)** Histological sections of the left ventricle stained with hematoxylin-eosin in each group. The arrows highlight the cellular nucleus for each group. **(B)** Quantitative analysis of mean nuclear area in all assessed groups. Data are represented as means ± SEM, (*n* = 3). ^∗∗^*p* < 0.01 vs. CTR, ^##^*p* < 0.01 vs. ISO, one-way ANOVA followed by Bonferroni’s post-test.

**FIGURE 5:**
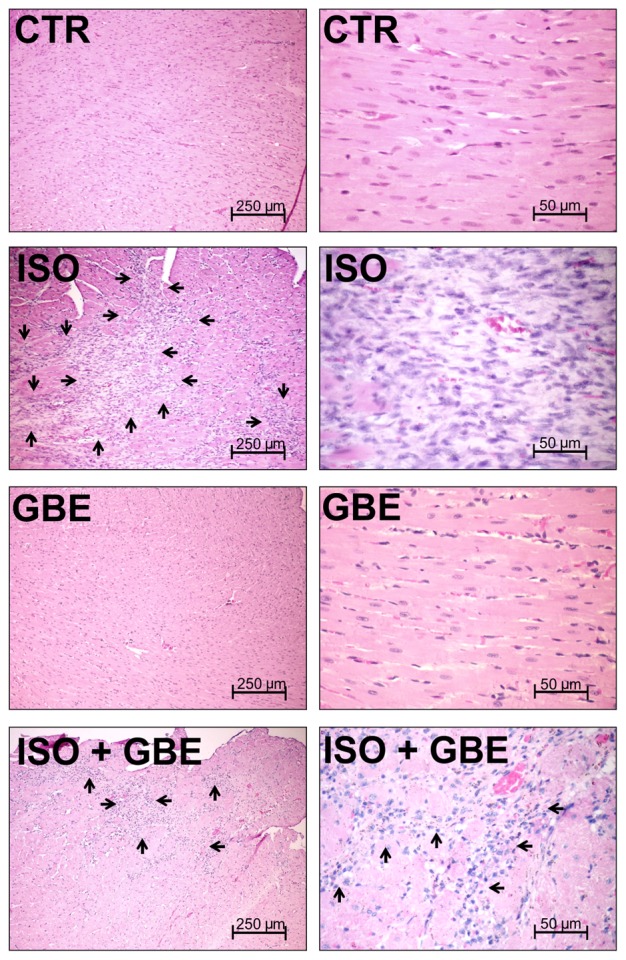
***Ginkgo biloba* extract prevents pathological myocardial remodeling induced by chronic β-adrenergic stimulation.** Histological sections of the left ventricle stained with hematoxylin-eosin in all of the groups evaluated. The CTR and *Ginkgo biloba* extract (GBE) groups demonstrate the cardiac striated muscle morphology. Areas of parenchymal replacement by fibrocellular connective tissue are highlighted by arrows. The ISO group demonstrates areas of parenchyma replacement by intense cardiac spindle cell proliferation (fibroblasts). Note that the ISO + GBE group presented a smaller area of parenchymal replacement by fibrocellular tissue (*n* = 3).

To confirm whether GBE treatment exhibits cardioprotection through impeding typical hypertrophic remodeling induced by chronic β-AR stimulation, morphometric analysis of nuclear cross-sectional area was evaluated. As shown in the **Figure 4**, left ventricular histological sections display increased nuclear area in ISO-treated rats, which was restored to the control condition with GBE treatment. GBE treatment alone had no effect on nuclear size. Moreover, ISO group showed areas that were characterized by intense spindle cell proliferation, identified as fibroblasts replacing the cardiac parenchyma (**Figure 5**). However, GBE treatment of ISO-treated rats exhibited markedly less expressive hypercellular areas, suggesting a minor replacement of cardiac parenchyma and therefore, a considerable decrease in the degree of cardiac remodeling. GBE-treated group demonstrated a typical cardiac parenchyma similar to untreated group, suggesting that the administration of GBE did not promote remarkable changes in the histological architecture of the heart.

### *Ginkgo biloba* Extract Prevents Electrocardiographic Changes and Restores the Impaired Left Ventricular Contractility Induced by Chronic β-AR Stimulation

**FIGURE 6:**
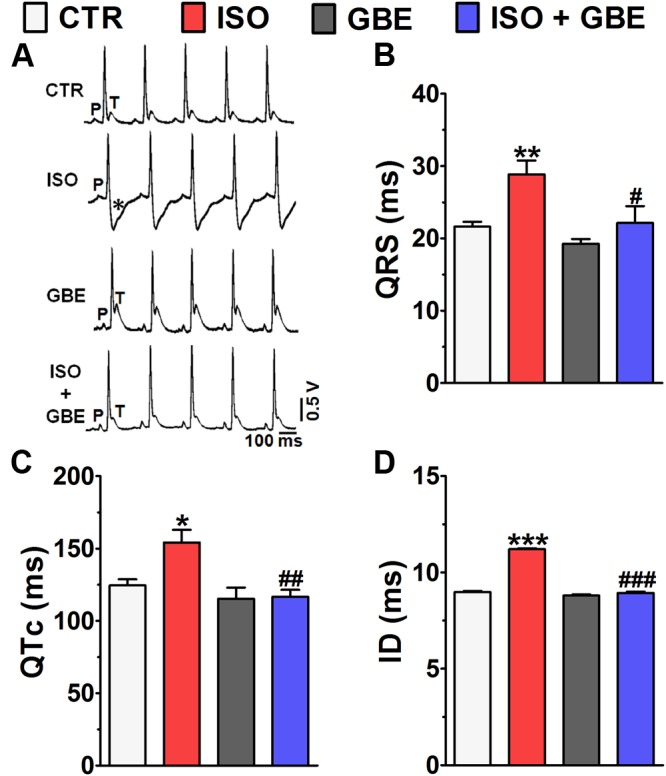
***Ginkgo biloba* extract prevents typical electrocardiographic changes of cardiac hypertrophy. (A)** Representative ECG recordings of four experimental groups. Asterisk indicates ST segment depression with a negative T-wave. Bar graph shows that GBE prevented increases in the **(B)** QRS complex, **(C)** QTc, and **(D)** intrinsicoid deflection (ID) interval. Data are represented as means ± SEM, (*n* = 14). ^∗^*p* < 0.05, ^∗∗^*p* < 0.01, and ^∗∗∗^*p* < 0.001 vs. CTR; ^#^*p* < 0.05, ^##^*p* < 0.01, and ^###^*p* < 0.001 vs. ISO, one-way ANOVA followed by Bonferroni’s Test.

Maladaptive cardiac remodeling is commonly associated with myocardial electric remodeling, therefore we next assessed whether GBE treatment prevents such electrical dysfunction through surface ECG recordings. **Figure 6A** shows typical ECG tracing in four experimental groups. It worthwhile note that depression of ST segment (negative T-wave) induced by chronic β-AR stimulation was abolished in GBE-treated rats. Moreover, rats that received ISO presented ECG changes that are typical of hypertrophic hearts such as, marked enlargement of QRS complex duration (**Figure 6B**), prolonged QTc interval (**Figure 6C**), and increased ID (**Figure 6D**) compared with control. Notably, concomitant treatment with GBE fully abolished all ECG changes found in ISO-treated rats (**Figures 6A–C**).

**FIGURE 7:**
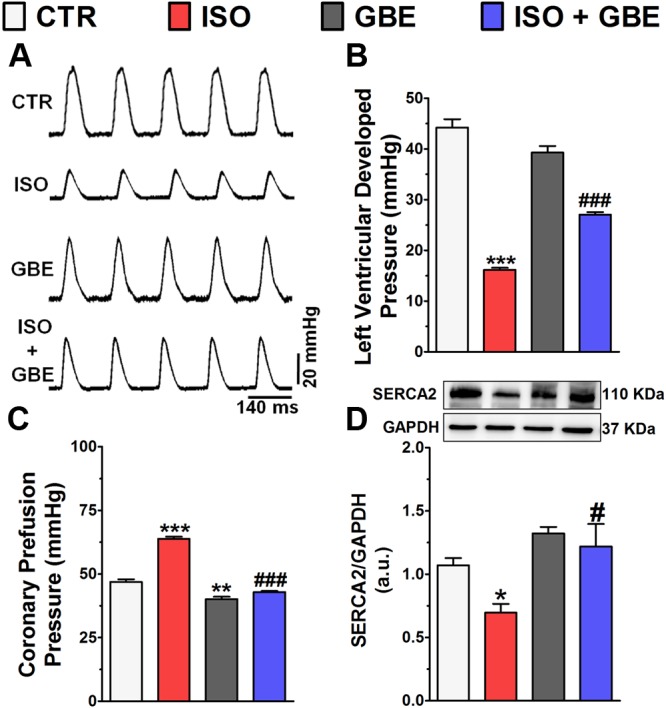
***Ginkgo biloba* extract restores the impaired ventricular contractility induced by chronic β-adrenergic stimulation. (A)** Representative left ventricular pressure recordings. Bar graph shows **(B)** left ventricular developed pressure measurements, **(C)** coronary perfusion pressure, **(D)** representative western blot and quantitative analysis of SERCA2. Data are represented as means ± SEM, (*n* = 6). ^∗^*p* < 0.05, ^∗∗^*p* < 0.01, and ^∗∗∗^*p* < 0.001 vs. CTR; ^#^*p* < 0.05 and ^###^*p* < 0.001 vs. ISO, one-way ANOVA followed by Bonferroni’s post-test.

Impaired myocardial contractility is a hallmark of pathological ventricular remodeling, therefore we also assessed whether GBE prevents the loss of ventricular contractility through Langendorff-isolated heart **Figure 7A**. As shown in the **Figure 7B**, chronic β-AR stimulation markedly decreased the LVDP when compared with control group, whereas GBE treatment significantly ameliorated the contractile dysfunction of hypertrophied hearts. GBE treatment alone did not affect the LVDP. Furthermore, during heart perfusion, the coronary perfusion pressure was simultaneously recorded in order to evaluate the coronary vasomotor tone. Chronic β-AR stimulation led to a higher coronary pressure when compared with control, whereas GBE treatment fully abolished this effect (**Figure 7C**). Interestingly, animals that only received GBE showed a reduced coronary pressure compared to untreated rats.

Abnormal Ca^2+^ handling is a common feature of impaired cardiac contractility, therefore it was evaluated the sarcoplasmic reticulum Ca^2+^ pump (SERCA2) expression in the heart. SERCA2 protein levels were significantly decreased in ventricles from ISO-treated rats compared to control (**Figure 7D**). In contrast, GBE treatment of ISO-treated rats fully restored the physiological levels of SERCA2.

### *Ginkgo biloba* Extract Prevents Cardiomyocyte Hypertrophy via M_2_/NO Pathway

**FIGURE 8:**
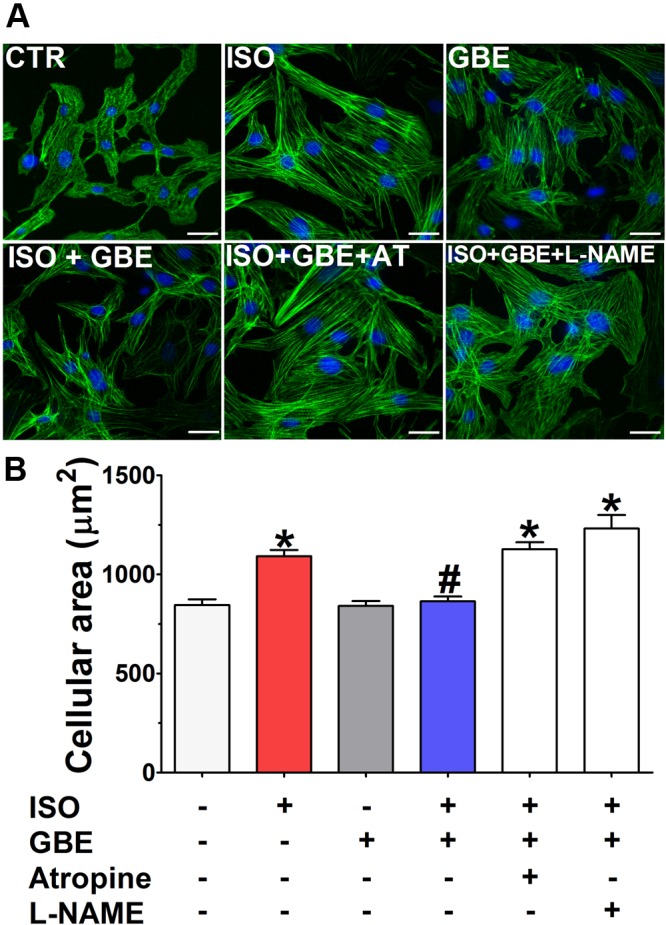
***Ginkgo biloba* extract suppresses cellular hypertrophy induced by isoproterenol *via* M_2_/NO pathway in rat neonatal cardiomyocytes. (A)** Representative immunofluorescence images from phalloidin/DAPI stained neonatal cardiomyocytes in control (CTR), *Ginkgo biloba* extract (GBE, 100 μg/mL), isoproterenol (ISO, 10 μM), isoproterenol plus *Ginkgo biloba* extract (ISO + GBE), isoproterenol plus *Ginkgo biloba* extract and muscarinic receptor antagonist, atropine (ISO + GBE + AT), isoproterenol plus *Ginkgo biloba* extract and nitric oxide synthase inhibitor, L-NAME (ISO + GBE + L-NAME) treated cells. **(B)** Quantification of cardiomyocyte surface area from experiments shown in **(A)**. Scale bar = 20 μm. Data are represented as means ± SEM, (*n* = 44–82 cells analyzed). ^∗^*p* < 0.05 vs. CTR and ^#^*p* < 0.05 vs. ISO, one-way ANOVA followed by Bonferroni’s post-test.

Taken together, our data suggest that the cardioprotective action of GBE involves the activation of cholinergic signaling. Therefore, to validate our findings, we next performed experiments on primary cultures of neonatal rat ventricular myocytes, which constitute a reliable *in vitro* model. Thus, in order to mimic the pathological conditions elicited by sustained β-AR stimulation, neonatal cardiomyocytes were treated with ISO and cellular hypertrophy was evaluated by measurement of myocyte surface area. As shown in the **Figure 8**, cardiomyocytes treated with ISO showed increased cell surface area, whereas co-treatment with GBE fully prevented this effect. Importantly, antihypertrophic action of GBE was abolished by atropine, a muscarinic receptor antagonist, or L-NAME, an inhibitor of NOS. Moreover, GBE alone had no effect on cellular area. Altogether, these data show that GBE antihypertrophic effect occurs *via* activation of M_2_/NO pathway.

## Discussion

The results of the present study demonstrate that GBE counteracts the deleterious cardiac actions of sustained β-AR activation by preventing autonomic imbalance, myocardial remodeling, aberrant ECG waveforms, and ventricular dysfunction. In addition, our findings unravel the activation of M_2_/NO pathway as a new mechanism involved in the remarkable antihypertrophic action of GBE.

Sympathovagal imbalance is observed during the development of several cardiovascular diseases (Shivkumar and Ardell, 2016). Accordingly, abnormal vagal activity is found during the early stage of cardiac dysfunction, whereas enhanced cholinergic activity has been associated with decreased pathological cardiac remodeling and risk of developing life-threatening arrhythmia (Li et al., 2004; Sabino et al., 2013). Therefore, pharmacological compounds that improve the cholinergic activity appear as a promising alternative therapy for cardiovascular disorders. In line with this hypothesis, our *in vivo* data show that GBE prevents the shift of cardiac autonomic balance toward a sympathetic drive and impaired baroreflex sensibility, as typically found in heart failure and hypertensive rats (Zucker et al., 1995; Botelho-Ono et al., 2011). Moreover, taking into account recent studies that have proposed the involvement of neuronal and non-neuronal cholinergic machinery in the protective effect against sustained β-AR stimulation (Rocha-Resende et al., 2012; Gavioli et al., 2014; Roy et al., 2016), in addition to the proposed cholinergic involvement in the GBE-mediated neuroprotection (Nathan, 2000; Kehr et al., 2012) we raised the hypothesis that GBE may promote cardioprotection against sustained β-AR stimulation *via* cholinergic pathway.

Stimulation of muscarinic receptors represents the primary trigger for activation of downstream intracellular signaling. Moreover, despite the well known five distinct muscarinic receptor subtypes (M_1_–M_5_), M_2_ receptor is the most abundant isoform in cardiomyocytes (Lara et al., 2010). Therefore, in the present study we focused on the M_2_ receptor due to the overwhelming expression in the heart. Here, we showed that M_2_ receptor was upregulated by chronic β-AR administration. This result correlated with previous *in vivo* and *in vitro* findings obtained from ISO-stimulated cardiomyocytes (Rocha-Resende et al., 2012; Gavioli et al., 2014). Importantly, GBE treatment prevented this change, indicating attenuated β-AR stimulation and therefore, the upregulation of M_2_ receptor was no longer required. Moreover, downregulation of β-AR has been consistently reported as a result of G protein-coupled receptor kinases and β-arrestin activities (Noor et al., 2011). In the present study, GBE treatment alone led to a downregulation of M_2_ and upregulation of β_1_-AR. In fact, our findings are strikingly similar to data obtained *in vivo* with rats subjected to treatment with pyridostigmine, a cholinesterase inhibitor (Gavioli et al., 2014). Along with these lines, previous studies have reported the anticholinesterase activity of GBE (Stein et al., 2015; Kim et al., 2016). Although the exact mechanism by which GBE alters cholinergic signaling is still unknown, it is plausible to assume that GBE acts, at least in part, through changes in cholinergic signaling.

Nitric oxide release has been reported as the downstream effector of cholinergic signaling in cardiomyocytes (Balligand et al., 1993; Rocha-Resende et al., 2012). Moreover, several studies have consistently demonstrated the beneficial actions of NO in the cardiovascular system by regulating eNOS and nNOS signaling pathways (Barouch et al., 2002; Ziolo et al., 2008; Umar and van der Laarse, 2009). *In vitro* and *in vivo* studies have shown that GBE enhances NO bioavailability (Sasaki et al., 2002; Koltermann et al., 2007). Accordingly, activation of NO signaling pathway by GBE treatment has been associated with cardioprotection in a model of ischaemia-reperfusion injury (Shen et al., 1998) and adriamycin-induced acute cardiotoxicity (El-Boghdady, 2013). In accordance with previous studies, we showed that despite increased eNOS expression in response to chronic β-AR stimulation (Champion et al., 2004; Krenek et al., 2009), its activity was markedly decreased, what would lead to reduced NO bioavailability (Victorio et al., 2016). The remarkable restoration of expression and impaired eNOS activity induced by GBE treatment of isoproterenol rats indicates eNOS-mediated NO as a negative modulator of chronic β-AR stimulation (Ozaki et al., 2002). Although recognized as a therapeutic target for several cardiovascular diseases (Zhang et al., 2014), in the present study, we showed that nNOS expression and activity remained unchanged upon chronic β-AR stimulation. Thereby these data rule out a significant contribution of nNOS-mediated NO in the antihypertrophic effect of GBE. Altogether, our data is the first evidence on the involvement of NO signaling pathway in the antihypertrophic effect of GBE.

Our data also endorse previous studies that correlate the diffuse myocardial damage characterized by fibroblast replacement with aberrant electrical remodeling (Tomaselli and Marbán, 1999; Chapman et al., 2001). Here, we show that GBE treatment prevented the pathological remodeling and ECG changes induced by chronic β-AR stimulation. Accordingly, reduced myocardial fibrosis induced by ligation of left anterior descending artery has been reported in GBE-treated rats. In this model, GBE treatment decreased expression levels of transforming growth factor-β1, and matrix metalloproteinase 2 and 9, therefore attenuating the extracellular matrix deposition (Li et al., 2015). The late-phase of hypertrophic remodeling is also associated with abnormalities in the Ca^2+^ handling, in which contributes to ventricular dysfunction (Bers et al., 2003; Lang et al., 2015). Altogether, our data indicate the GBE attenuates the decrease of ventricular wall compliance and increase of stiffness, thereby ameliorating the ventricular dysfunction. In addition, decreased expression of the SERCA2 was found in ISO-treated rats. This finding is consistent with reduced sarcoplasmic reticulum Ca^2+^ load, already described during cardiac hypertrophy stage, thereby affecting SR Ca^2+^ refilling and, consequently, ventricular contractility (Bers et al., 2003; Gavioli et al., 2014). Therefore, restoration of SERCA2 levels in GBE-treated rats represents an important underlying mechanism involved in the ventricular dysfunction amelioration.

β-adrenergic receptors are also expressed on the endothelial and smooth muscle cells and, its actions seems to be dependent of the vascular bed and isoforms (Flacco et al., 2013). In resistance artery, chronic β-AR stimulation leads to impaired vascular tone (Davel et al., 2006), increased expression of proinflammatory cytokines and NF-κB activity (Davel et al., 2008), and decreased NO bioavailability (Victorio et al., 2016). Accordingly, in the present study, we demonstrated that chronic β-AR stimulation caused a marked increase in the coronary resistance, which was fully prevented by GBE treatment. Moreover, we showed the rats treated with GBE displayed lower coronary perfusion pressure. Supporting our findings, the vasodilator activity of GBE was previously demonstrated in rat aorta rings through opening of Ca^2+^-activated potassium channel, in which ultimately causes endothelial cell hyperpolarization and NO release by eNOS activity (Satoh and Nishida, 2004).

Although our data indicate that GBE treatment counteracts the deleterious cardiac actions of sustained β-AR stimulation *via* cholinergic pathway, we unequivocally validated our hypothesis through *in vitro* experiments. Accordingly, we showed the antihypertrophic action of GBE was fully abolished by either, muscarinic receptor or NOS inhibition. Indeed, the downstream cascade of muscarinic pathway involves the activation of NOS and, consequently, NO release (Rocha-Resende et al., 2012). Consistent with our data, previous study demonstrated that similar dose of GBE (100 μg/mL) enhanced NO production by increasing eNOS activity in endothelial cells (Koltermann et al., 2007). Moreover, it is worthy of note that the *in vitro* concentration used in the present study was determined in accordance with pharmacokinetics studies in human that have demonstrated this concentration is likely to be achieved in the blood after daily intake of 80–240 mg, which represents the regular dosage of GBE for effective therapy (Biber, 2003; Ude et al., 2013). Altogether, our results bring new insights into the mechanism involved in the antihypertrophic action of GBE, which goes beyond its antioxidant activity.

## Conclusion

In summary, our data show that the deleterious cardiac actions of sustained β-AR activation were significantly attenuated by GBE treatment. Furthermore, our findings indicate that pharmacological actions of GBE treatment alone on sympathetic-cholinergic receptors may be involved in the cardioprotective effect. Accordingly, we show that the antihypertrophic action of GBE occurs *via* activation of M_2_/NO pathway. Overall, these findings uncover a new mechanism involved in the cardioprotective action of GBE.

## Author Contributions

TM and IdJ participated in all steps of this study. JdS, GdA, FM, RdS, JdM-F, RM-d-S, PM, SS, and RP-F performed experiments. CdV, SG, VS-F, RA-J, and SL-S contributed to the experimental design, data analyses, data interpretation, and the preparation and revision of the manuscript.

## Conflict of Interest Statement

The authors declare that the research was conducted in the absence of any commercial or financial relationships that could be construed as a potential conflict of interest. The reviewer CASP and handling Editor declared their shared affiliation, and the handling Editor states that the process nevertheless met the standards of a fair and objective review.

## Footnotes

1 http://sites.google.com/site/cardioseries

## References

[B1] Balligand J. L. Kelly R. A. Marsden P. A. Smith T. W. Michel T. (1993). Control of cardiac muscle cell function by an endogenous nitric oxide signaling system. *Proc. Natl. Acad. Sci. U.S.A.* 90 347–351. 10.1073/pnas.90.1.347 7678347 PMC45657

[B2] Barouch L. A. Harrison R. W. Skaf M. W. Rosas G. O. Cappola T. P. Kobeissi Z. A. (2002). Nitric oxide regulates the heart by spatial confinement of nitric oxide synthase isoforms. *Nature* 416 337–339. 10.1038/416337a 11907582

[B3] Bers D. M. Eisner D. A. Valdivia H. H. (2003). Sarcoplasmic reticulum Ca^2+^ and heart failure. *Circ. Res.* 93 487–490. 10.1161/01.RES.0000091871.54907.6B 14500331

[B4] Bertinieri G. di Rienzo M. Cavallazzi A. Ferrari A. U. Pedotti A. Mancia G. (1985). A new approach to analysis of the arterial baroreflex. *J. Hypertens. Suppl.* 3 S79–S81. 2856787

[B5] Biber A. (2003). Pharmacokinetics of *Ginkgo biloba* extracts. *Pharmacopsychiatry* 36(Suppl. 1) S32–S37. 10.1055/s-2003-40446 13130386

[B6] Botelho-Ono M. S. Pina H. V. Sousa K. H. F. Nunes F. C. Medeiros I. A. Braga V. A. (2011). Acute superoxide scavenging restores depressed baroreflex sensitivity in renovascular hypertensive rats. *Auton. Neurosci.* 159 38–44. 10.1016/j.autneu.2010.07.025 20719579

[B7] Champion H. C. Georgakopoulos D. Takimoto E. Isoda T. Wang Y. Kass D. A. (2004). Modulation of in vivo cardiac function by myocyte-specific nitric oxide synthase-3. *Circ. Res.* 94 657–663. 10.1161/01.RES.0000119323.79644.20 14752030

[B8] Chapman N. Mayet J. Ozkor M. Lampe F. C. Thom S. A. Poulter N. R. (2001). QT intervals and QT dispersion as measures of left ventricular hypertrophy in an unselected hypertensive population. *Am. J. Hypertens.* 14 455–462. 10.1016/S0895-7061(00)01292-9 11368467

[B9] Chou T. C. Yen M. H. Li C. Y. Ding Y. A. (1998). Alterations of nitric oxide synthase expression with aging and hypertension in rats. *Hypertension* 1979 643–648. 10.1161/01.HYP.31.2.643 9461235

[B10] Couto G. K. Britto L. R. G. Mill J. G. Rossoni L. V. (2015). Enhanced nitric oxide bioavailability in coronary arteries prevents the onset of heart failure in rats with myocardial infarction. *J. Mol. Cell. Cardiol.* 86 110–120. 10.1016/j.yjmcc.2015.07.017 26225841

[B11] Davel A. P. C. Fukuda L. E. Sá L. L. D. Munhoz C. D. Scavone C. Sanz-Rosa D. (2008). Effects of isoproterenol treatment for 7 days on inflammatory mediators in the rat aorta. *Am. J. Physiol. Heart Circ. Physiol.* 295 H211–H219. 10.1152/ajpheart.00581.2007 18487443

[B12] Davel A. P. C. Kawamoto E. M. Scavone C. Vassallo D. V. Rossoni L. V. (2006). Changes in vascular reactivity following administration of isoproterenol for 1 week: a role for endothelial modulation. *Br. J. Pharmacol.* 148 629–639. 10.1038/sj.bjp.0706749 16702995 PMC1751879

[B13] du Toit E. F. Genade S. Carlini S. Moolman J. A. Brunner F. Lochner A. (2007). Efficacy of ischaemic preconditioning in the eNOS overexpressed working mouse heart model. *Eur. J. Pharmacol.* 556 115–120. 10.1016/j.ejphar.2006.11.004 17157294

[B14] El-Boghdady N. A. (2013). Increased cardiac endothelin-1 and nitric oxide in adriamycin-induced acute cardiotoxicity: protective effect of *Ginkgo biloba* extract. *Indian J. Biochem. Biophys.* 50 202–209. 23898483

[B15] Flacco N. Segura V. Perez-Aso M. Estrada S. Seller J. Jiménez-Altayó F. (2013). Different β-adrenoceptor subtypes coupling to cAMP or NO/cGMP pathways: implications in the relaxant response of rat conductance and resistance vessels. *Br. J. Pharmacol.* 169 413–425. 10.1111/bph.12121 23373597 PMC3651666

[B16] Gavioli M. Lara A. Almeida P. W. M. Lima A. M. Damasceno D. D. Rocha-Resende C. (2014). Cholinergic signaling exerts protective effects in models of sympathetic hyperactivity-induced cardiac dysfunction. *PLoS ONE* 9:e100179 10.1371/journal.pone.0100179 PMC4081111 24992197

[B17] Kehr J. Yoshitake S. Ijiri S. Koch E. Nöldner M. Yoshitake T. (2012). *Ginkgo biloba* leaf extract (EGb 761^®^) and its specific acylated flavonol constituents increase dopamine and acetylcholine levels in the rat medial prefrontal cortex: possible implications for the cognitive enhancing properties of EGb 761^®^. *Int. Psychogeriatr.* 24(Suppl. 1) S25–S34. 10.1017/S1041610212000567 22784425

[B18] Kim M.-S. Bang J. H. Lee J. Han J.-S. Baik T. G. Jeon W. K. (2016). *Ginkgo biloba* L. extract protects against chronic cerebral hypoperfusion by modulating neuroinflammation and the cholinergic system. *Phytomedicine* 23 1356–1364. 10.1016/j.phymed.2016.07.013 27765355

[B19] Koltermann A. Hartkorn A. Koch E. Fürst R. Vollmar A. M. Zahler S. (2007). *Ginkgo biloba* extract EGb^®^ 761 increases endothelial nitric oxide production *in vitro* and *in vivo*. *Cell. Mol. Life Sci.* 64 1715–1722. 10.1007/s00018-007-7085-z 17497242 PMC11136141

[B20] Krenek P. Kmecova J. Kucerova D. Bajuszova Z. Musil P. Gazova A. (2009). Isoproterenol-induced heart failure in the rat is associated with nitric oxide-dependent functional alterations of cardiac function. *Eur. J. Heart Fail.* 11 140–146. 10.1093/eurjhf/hfn026 19168511 PMC2639419

[B21] Kubota Y. Tanaka N. Kagota S. Nakamura K. Kunitomo M. Umegaki K. (2006). Effects of *Ginkgo biloba* extract on blood pressure and vascular endothelial response by acetylcholine in spontaneously hypertensive rats. *J. Pharm. Pharmacol.* 58 243–249. 10.1211/jpp.58.2.0012 16451753

[B22] Kuller L. H. Ives D. G. Fitzpatrick A. L. Carlson M. C. Mercado C. Lopez O. L. (2010). Does *Ginkgo biloba* reduce the risk of cardiovascular events? *Circ. Cardiovasc. Qual. Outcomes* 3 41–47. 10.1161/CIRCOUTCOMES.109.871640 20123670 PMC2858335

[B23] Lang D. Holzem K. Kang C. Xiao M. Hwang H. J. Ewald G. A. (2015). Arrhythmogenic remodeling of β2 versus β1 adrenergic signaling in the human failing heart. *Circ. Arrhythm. Electrophysiol.* 8 409–419. 10.1161/CIRCEP.114.002065 25673629 PMC4608687

[B24] Lara A. Damasceno D. D. Pires R. Gros R. Gomes E. R. Gavioli M. (2010). Dysautonomia due to reduced cholinergic neurotransmission causes cardiac remodeling and heart failure. *Mol. Cell. Biol.* 30 1746–1756. 10.1128/MCB.00996-09 20123977 PMC2838086

[B25] Li M. Zheng C. Sato T. Kawada T. Sugimachi M. Sunagawa K. (2004). Vagal nerve stimulation markedly improves long-term survival after chronic heart failure in rats. *Circulation* 109 120–124. 10.1161/01.CIR.0000105721.71640.DA 14662714

[B26] Li W. Luo Z. Liu X. Fu L. Xu Y. Wu L. (2015). Effect of *Ginkgo biloba* extract on experimental cardiac remodeling. *BMC Complement. Altern. Med.* 15:277 10.1186/s12906-015-0719-z PMC4534054 26268459

[B27] Liebgott T. Miollan M. Berchadsky Y. Drieu K. Culcasi M. Pietri S. (2000). Complementary cardioprotective effects of flavonoid metabolites and terpenoid constituents of *Ginkgo biloba* extract (EGb 761) during ischemia and reperfusion. *Basic Res. Cardiol.* 95 368–377. 10.1007/s003950070035 11099163

[B28] Liu T.-J. Yeh Y.-C. Ting C.-T. Lee W.-L. Wang L.-C. Lee H.-W. (2008). *Ginkgo biloba* extract 761 reduces doxorubicin-induced apoptotic damage in rat hearts and neonatal cardiomyocytes. *Cardiovasc. Res.* 80 227–235. 10.1093/cvr/cvn192 18632596

[B29] Macedo F. N. Mesquita T. R. R. Melo V. U. Mota M. M. Silva T. L. T. B. Santana M. N. (2016). Increased nitric oxide bioavailability and decreased sympathetic modulation are involved in vascular adjustments induced by low-intensity resistance training. *Front. Physiol.* 7:265 10.3389/fphys.2016.00265 PMC4923192 27445854

[B30] Mota M. M. Mesquita T. R. R. Braga da Silva T. L. T. Fontes M. T. Lauton Santos S. dos Santos Aggum Capettini L. (2015). Endothelium adjustments to acute resistance exercise are intensity-dependent in healthy animals. *Life Sci.* 142 86–91. 10.1016/j.lfs.2015.10.007 26455551

[B31] Nakata S. Tsutsui M. Shimokawa H. Suda O. Morishita T. Shibata K. (2008). Spontaneous myocardial infarction in mice lacking all nitric oxide synthase isoforms. *Circulation* 117 2211–2223. 10.1161/CIRCULATIONAHA.107.742692 18413498

[B32] Nathan P. (2000). Can the cognitive enhancing effects of *ginkgo biloba* be explained by its pharmacology? *Med. Hypotheses* 55 491–493. 10.1054/mehy.2000.1099 11090296

[B33] Noor N. Patel C. B. Rockman H. A. (2011). β-Arrestin: a signaling molecule and potential therapeutic target for heart failure. *J. Mol. Cell. Cardiol.* 51 534–541. 10.1016/j.yjmcc.2010.11.005 21074538 PMC3063861

[B34] Ozaki M. Kawashima S. Yamashita T. Hirase T. Ohashi Y. Inoue N. (2002). Overexpression of endothelial nitric oxide synthase attenuates cardiac hypertrophy induced by chronic isoproterenol infusion. *Circ. J.* 66 851–856. 10.1253/circj.66.851 12224825

[B35] Panda V. S. Naik S. R. (2008). Cardioprotective activity of *Ginkgo biloba* phytosomes in isoproterenol-induced myocardial necrosis in rats: a biochemical and histoarchitectural evaluation. *Exp. Toxicol. Pathol.* 60 397–404. 10.1016/j.etp.2008.03.010 18513933

[B36] Pietri S. Maurelli E. Drieu K. Culcasi M. (1997). Cardioprotective and anti-oxidant effects of the terpenoid constituents of *Ginkgo biloba* extract (EGb 761). *J. Mol. Cell. Cardiol.* 29 733–742. 10.1006/jmcc.1996.0316 9140830

[B37] Rocha-Resende C. Roy A. Resende R. Ladeira M. S. Lara A. de Morais Gomes E. R. (2012). Non-neuronal cholinergic machinery present in cardiomyocytes offsets hypertrophic signals. *J. Mol. Cell. Cardiol.* 53 206–216. 10.1016/j.yjmcc.2012.05.003 22587993 PMC3806714

[B38] Roy A. Dakroub M. Tezini G. C. S. V. Liu Y. Guatimosim S. Feng Q. (2016). Cardiac acetylcholine inhibits ventricular remodeling and dysfunction under pathologic conditions. *FASEB J.* 30 688–701. 10.1096/fj.15-277046 26481308 PMC6188225

[B39] Sabino J. P. J. da Silva C. A. A. de Melo R. F. Fazan R. Jr. Salgado H. C. (2013). The treatment with pyridostigmine improves the cardiocirculatory function in rats with chronic heart failure. *Auton. Neurosci.* 173 58–64. 10.1016/j.autneu.2012.11.007 23218833

[B40] Sasaki Y. Noguchi T. Yamamoto E. Giddings J. C. Ikeda K. Yamori Y. (2002). Effects of *Ginkgo biloba* extract (EGb 761) on cerebral thrombosis and blood pressure in stroke-prone spontaneously hypertensive rats. *Clin. Exp. Pharmacol. Physiol.* 29 963–967. 10.1046/j.1440-1681.2002.03761.x 12366386

[B41] Satoh H. Nishida S. (2004). Electropharmacological actions of *Ginkgo biloba* extract on vascular smooth and heart muscles. *Clin. Chim. Acta* 342 13–22. 10.1016/j.cccn.2003.12.014 15026263

[B42] Shen J. Wang J. Zhao B. Hou J. Gao T. Xin W. (1998). Effects of EGb 761 on nitric oxide and oxygen free radicals, myocardial damage and arrhythmia in ischemia-reperfusion injury in vivo. *Biochim. Biophys. Acta* 1406 228–236. 10.1016/S0925-4439(98)00007-6 9630646

[B43] Shivkumar K. Ardell J. L. (2016). Cardiac autonomic control in health and disease. *J. Physiol.* 594 3851–3852. 10.1113/JP272580 27417670 PMC4945710

[B44] Stein C. Hopfeld J. Lau H. Klein J. (2015). Effects of *Ginkgo biloba* extract EGb 761, donepezil and their combination on central cholinergic function in aged rats. *J. Pharm. Pharm. Sci.* 18 634–646. 10.18433/J3WC8V 26626253

[B45] Tan M.-S. Yu J.-T. Tan C.-C. Wang H.-F. Meng X.-F. Wang C. (2015). Efficacy and adverse effects of *ginkgo biloba* for cognitive impairment and dementia: a systematic review and meta-analysis. *J. Alzheimers Dis.* 43 589–603. 10.3233/JAD-140837 25114079

[B46] Tomaselli G. F. Marbán E. (1999). Electrophysiological remodeling in hypertrophy and heart failure. *Cardiovasc. Res.* 42 270–283. 10.1016/S0008-6363(99)00017-6 10533566

[B47] Trumbeckaite S. Bernatoniene J. Majiene D. Jakstas V. Savickas A. Toleikis A. (2007). Effect of *Ginkgo biloba* extract on the rat heart mitochondrial function. *J. Ethnopharmacol.* 111 512–516. 10.1016/j.jep.2006.12.028 17258877

[B48] Ude C. Schubert-Zsilavecz M. Wurglics M. (2013). *Ginkgo biloba* extracts: a review of the pharmacokinetics of the active ingredients. *Clin. Pharmacokinet.* 52 727–749. 10.1007/s40262-013-0074-5 23703577

[B49] Umar S. van der Laarse A. (2009). Nitric oxide and nitric oxide synthase isoforms in the normal, hypertrophic, and failing heart. *Mol. Cell. Biochem.* 333 191–201. 10.1007/s11010-009-0219-x 19618122

[B50] Victorio J. A. Clerici S. P. Palacios R. Alonso M. J. Vassallo D. V. Jaffe I. Z. (2016). Spironolactone prevents endothelial nitric oxide synthase uncoupling and vascular dysfunction induced by β-adrenergic OverstimulationNovelty and significance. *Hypertension* 68 726–735. 10.1161/HYPERTENSIONAHA.116.07911 27432866 PMC4978608

[B51] Wang H. Kohr M. J. Wheeler D. G. Ziolo M. T. (2008). Endothelial nitric oxide synthase decreases β-adrenergic responsiveness via inhibition of the L-type Ca^2+^ current. *Am. J. Physiol. Heart Circ. Physiol.* 294 H1473–H1480. 10.1152/ajpheart.01249.2007 18203845 PMC2744450

[B52] Wang Z. Zhang J. Ren T. Dong Z. (2016). Targeted metabolomic profiling of cardioprotective effect of *Ginkgo biloba* L. extract on myocardial ischemia in rats. *Phytomedicine* 23 621–631. 10.1016/j.phymed.2016.03.005 27161403

[B53] Yoshikawa T. Naito Y. Kondo M. (1999). *Ginkgo biloba* leaf extract: review of biological actions and clinical applications. *Antioxid. Redox Signal.* 1 469–480. 10.1089/ars.1999.1.4-469 11233145

[B54] Zhang Y. H. Jin C. Z. Jang J. H. Wang Y. (2014). Molecular mechanisms of neuronal nitric oxide synthase in cardiac function and pathophysiology. *J. Physiol.* 592 3189–3200. 10.1113/jphysiol.2013.270306 24756636 PMC4146369

[B55] Zhou W. Chai H. Lin P. H. Lumsden A. B. Yao Q. Chen C. (2004). Clinical use and molecular mechanisms of action of extract of *Ginkgo biloba* leaves in cardiovascular diseases. *Cardiovasc. Drug Rev.* 22 309–319. 10.1111/j.1527-3466.2004.tb00148.x 15592576

[B56] Ziolo M. T. Kohr M. J. Wang H. (2008). Nitric oxide signaling and the regulation of myocardial function. *J. Mol. Cell. Cardiol.* 45 625–632. 10.1016/j.yjmcc.2008.07.015 18722380 PMC3282562

[B57] Zucker I. H. Wang W. Brändle M. Schultz H. D. Patel K. P. (1995). Neural regulation of sympathetic nerve activity in heart failure. *Prog. Cardiovasc. Dis.* 37 397–414. 10.1016/S0033-0620(05)80020-9 7777669

